# The biological characteristics and life table parameters of *Plodia interpunctella* (Lepidoptera: Pyralidae) reared on different maize varieties

**DOI:** 10.1093/jisesa/ieaf047

**Published:** 2025-05-16

**Authors:** Zahra Saeedi, Masumeh Ziaee, Mehdi Esfandiari, Somaiyeh Ghasemzadeh

**Affiliations:** Department of Plant Protection, Faculty of Agriculture, Shahid Chamran University of Ahvaz, Ahvaz, Iran; Department of Plant Protection, Faculty of Agriculture, Shahid Chamran University of Ahvaz, Ahvaz, Iran; Department of Plant Protection, Faculty of Agriculture, Shahid Chamran University of Ahvaz, Ahvaz, Iran; Entomology Research Laboratory, University of Vermont, Burlington, VT, USA

**Keywords:** Indianmeal moth, life span, fecundity, growth, stored-grain insect pests

## Abstract

The Indianmeal moth, *Plodia interpunctella* (Hübner) is a major polyphagous pest of stored food products causing serious quantity and quality losses. In this study, the life history of *P. interpunctella* was evaluated on different maize varieties, including Simon, Valbom, 703, BK, and BC678. The preadult duration for *P. interpunctella* were 35.5, 43.1, 39.2, 43.4, and 36.8 d on Simon, Valbom, 703, BK, and BC678, respectively. The mean total longevity on Valbom was 52.2 d which was significantly longer than the 41.8 d on Simon (*P* = 0.012). The developmental period of moths was the most prolonged on Valbom indicating low nutritional suitability of this variety. However, the moths preferred Valbom for oviposition, and more than 132 eggs were laid on this variety. The most intrinsic rate of increase (*r*) was reported on BC678 and Simon, while the lowest population growth rate was on BK and Valbom. The highest *r* value on BC678 and Simon could be due to their high moisture and protein content. Based on the shorter preadult, total preoviposition period, mean generation time and higher life table parameters (gross reproductive rate, *r*, and *λ*) that occurred on the BC678, make this variety most favorable host for *P. interpunctella*. The findings highlighted the importance of maize variety selection in managing this pest in stored food products.

## Graphical Abstract

**Graphical Abstract:**
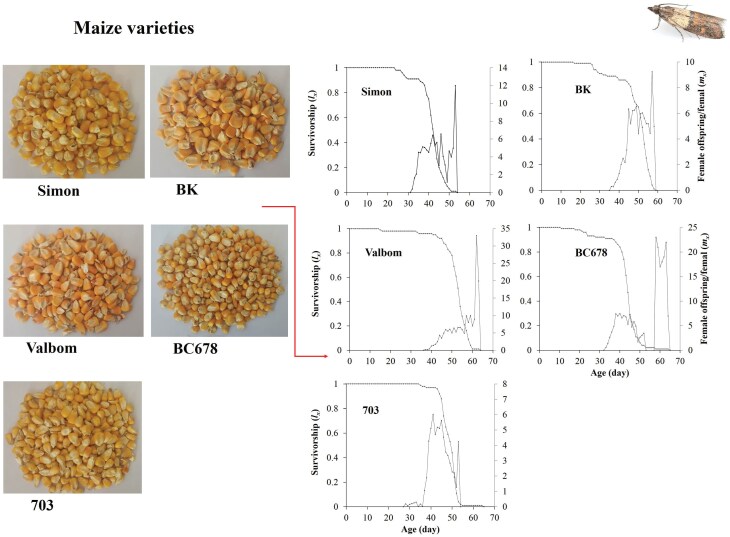

Maize, *Zea mays* L., is one of the most important and strategic products worldwide. It is the second widely grown crop after wheat, and cultivated on about 197 M ha lands in the world with approximately 1,137 million tons annual production in 2019 (Erenstein et al. 2022). Khuzestan province ranks third with 11.32% maize production of total maize producers in Iran (Ahmadi et al. 2022). Maize is an annual plant belonging to the monocotyledon class, and the tribe Andropogoneae, Panicoideae subfamily, and family Poaceae (Awata et al. 2019). All parts of the maize plant including seeds, stems, leaves, and even its cob are used for different purposes as a nutritional source for human, livestock and poultry. Maize has been considered a raw material in various industries (Shiferaw et al. 2011).

One of the important goals of sustainable agriculture is to produce healthy and high-quality products to increase economic profit and supply them to the market. Many problems prevent the achievement of these goals. One is the presence and activity of stored-products insect pests and their damage to food commodities. Infestations of pests cause significant quantitative and qualitative losses during storage (Gamage et al. 2023). The chemical composition, color, and taste of the products are changed because of the damage caused by storage pests. Their commercial and consumer value is significantly reduced, and sometimes they become entirely unusable (Kumar and Kalita 2017, Stathas et al. 2023).

Insect pests mostly damage foods stored in bags and bins which can cause extensive post-harvest losses (Ahmad et al. 2021). One of the important storage pests is the Indianmeal moth, *Plodia interpunctella* (Hübner) (Lepidoptera: Pyralidae). This moth is a worldwide pest, and causes severe damage to various processed food commodities in storages, and warehouses (Mohandass et al. 2007). The main damage is inflicted by larvae, which feed on cereals, dried fruits, nuts, oilseeds, and vegetables in storage (Hill 2002, Mohandass et al. 2007). There are several reports on the presence and activity of *P. interpunctella* on stored maize (Abdel-Rahman et al. 1968, Mbata 1990, Arbogast 2007, Throne and Arbogast 2010, Predojević et al. 2017). The larvae primarily feed on the germinal part of maize kernels causing loss of seed viability and reducing the germination rate. The quality of maize is destroyed due to contamination with silk and larval feces. The larvae of *P. interpunctella* had higher development and growth on broken kernels. This preference highlights the importance of grain integrity in pest management (Predojević et al. 2017). The impact of *P. interpunctella* extends beyond mere physical damage; it also poses challenges for food security and agricultural sustainability. Infestations can lead to financial losses for farmers and contribute to food scarcity in affected regions. Therefore, understanding the biology and behavior of this pest is crucial for developing effective management strategies growth (Doud and Phillips 2000, Mohandass et al. 2007, Mishra et al. 2024).

Different maize varieties may exhibit varying levels of susceptibility to insect’s infestation, making it essential to evaluate the life history traits, reproductive capacity, and overall life table parameters of *P. interpunctella* across diverse maize varieties. The current study aimed to compare these parameters across 5 stored maize varieties: Simon, Valbom, 703, BK, and BC678. By categorizing these varieties based on their suitability to *P. interpunctella*, this research intends to provide valuable insights that can inform the design of targeted management programs. Effective pest management strategies are critical for minimizing post-harvest losses and ensuring the quality of stored maize. Through a comprehensive understanding of the interactions between *P. interpunctella* and different maize varieties, we can develop sustainable practices that protect both agricultural productivity and food quality.

## Materials and Methods

### Maize Varieties

Commercial maize varieties, including Simon, Valbom, 703, BK, and BC678 were sourced from Iranian Company for Maize Development, Dezful, Khuzestan province. To eliminate any existing insect infestations, the maize kernels were stored at −15 °C for 2 wk. Following this cold treatment, the kernels were placed in an incubator at 26 ± 1 °C with a relative humidity (RH) of 60 ± 5% for 1 wk to achieve the moisture content (m.c.) necessary for the experimental conditions (Adarkwah et al. 2022).

### Insect Rearing

The initial colony of *P. interpunctella* was collected from stored maize in warehouses of Khuzestan province during June 2022. They were separately reared on selected maize varieties inside plastic cylinder containers (1 liter) at 26 ± 1 °C, 60 ± 5% RH, and a photoperiod of 16 h:8 h (L:D). Adults of the third generation were used for the experiments.

### The Biological Characteristics and Life Table Parameters of *P. interpunctella* on Different Maize Varieties

The biological characteristics and life table parameters of *P. interpunctella* were evaluated on 5 different maize varieties. A pair of adults (male and female) was released in each plastic container (80 ml), containing 10 g maize kernels of each variety. Plastic Petri dishes (9 cm in diameter and 1.5 cm in height) were used for the experiments. A valve (3 cm diameter) was created in the middle of the lid of each dish and covered with a fine mesh cloth to provide ventilation. After 24 h, the laid eggs were removed from pericarp of maize kernels and transferred to Petri dishes (each containing one egg). The Petri dishes were placed in an incubator at 26 ± 1 °C, 60 ± 5% RH, and 16 h:8 h (L: D) photoperiod. For each maize variety, 100 Petri dishes were utilized. The eggs were examined daily under a stereomicroscope, and larval hatching was recorded. The newly hatched larvae were transferred individually to dishes containing maize kernels (10 g) for the feeding. Maize kernels were placed in the Petri dishes daily, and the development and mortality of larvae were checked daily until they reached the pupal stage. The gender of larvae was determined; male larvae have a black spot in the medium plane of the mid-dorsal abdomen segment (Allotey and Goswami 1990). The Petri dishes were checked every 24 h until adult’s emergence. Then, male and female moths were paired on each variety separately in the plastic containers, and a cotton wool pad moistened with 10% honey solution was provided as adult’s food. The number of eggs laid was counted daily until the death of the female.

### Characteristics of Maize Kernels

#### Moisture Content

To determine the m.c. of maize kernels, 10 g of maize from each variety was milled in triplicate, then dried in an oven set at 110 °C. Dry weight was measured, and moisture percentage was determined (Delgarm et al. 2020).

#### Protein Content

The protein content of maize kernels was determined using Bradford’s reagent. The Bradford reagent was prepared by adding 100 mg CBBG 250 (Coomassie Brilliant blue G 250) dye, 50 ml 95% ethanol, and 100 ml phosphoric acid 85%. The solution was mixed until the blue dye was dissolved and reached a total volume of 1,000 ml. The standard curve and the equation of the calibration curve (*y* = 0.002*x* + 0.014) were generated by plotting the average blank corrected 595 nm measurement for each standard versus its concentration. For the standard solution preparation, 100 mg BSA (Bovine Serum Albumin) was dissolved in 50 ml ethanol and 100 m phosphoric acid 85%. It was then diluted with ultrapure water to reach a total volume of 1,000 ml. Maize kernels were ground, and 1 g of the sample was mixed with 10 ml normal sodium hydroxide 1 M for 5 min. The solution was centrifuged (30,000 × *g*, 10 min at 2 °C), and 1 ml of the supernatant was mixed with 2 ml Bradford reagent and stirred for 15 min. Then, 2 ml of the solution was added to the tube containing 3 ml Bradford’s reagent and 5 ml of distilled water was added and mixed thoroughly. The tubes were incubated in the dark at room temperature for 15 min. The absorbance was determined at 595 nm using a UV–Vis spectrophotometer (Bradford 1976). Three replicates were performed for each variety.

#### Starch Content

The starch content of maize kernels was determined using anthrone reagent with 3 replicates. Maize kernels were ground, and 100 mg of the sample was mixed with 2 ml of 80% ethanol, and incubated in a water bath at 80 °C for 30 min, followed by centrifuging at 3,000 × *g* for 10 min. The supernatant was removed to a tube. Then, 2 ml of HCIO_4_ (9.2 mol/liter) was added to the precipitate while stirring for 15 min. After that 4 ml of ultrapure water was added to the solution and mixed for 15 min. The mixture was centrifuged at 3,000 × *g* for 10 min, and the supernatant was transferred to a new tube. Subsequently, 2 ml of HCIO_4_ (4.6 mol/liter) was added to the precipitate while stirring for 15 min, then 5 ml of ultrapure water was added to the solution and mixed for 15 min. The mixture was centrifuged at 3,000 × *g* for 10 min, and the supernatant was added to the previous tube and mixed with supernatant solutions. The precipitate was washed twice with ultrapure water. The anthrone–sulfuric acid solution was prepared by adding 0.2 g of anthrone and 1.0 g of thiourea dissolved in 100 ml concentrated sulfuric acid and incubated in 100 °C water baths for 10 min. Then, 2 ml of supernatant mixture was added to 5 ml of an anthrone–sulfuric acid solution. The absorbance was determined at 630 nm using a UV–Vis spectrophotometer (UNICO-2100, New Jersey, USA). The standard curve was constructed with the absorbance values of solutions containing different glucose concentrations (Zhou et al. 2023).

### Statistical Analysis

The life history and demographic parameters of *P. interpunctella* were analyzed based on the age–stage, 2-sex life table (Chi and Liu 1985, Chi 1988), using TWOSEX MS Chart software (Chi 2022). The age–stage specific survival rate (*s*_*xj*_), age–stage specific reproductive value (*v*_*xj*_), and age–stage specific life expectancy (*e*_*xj*_) (where *x* = age and *j* = stage) were calculated (Chi and Su 2006). The mean and standard error of life table parameters were estimated using the Bootstrap method with 100,000 repetitions. (Efron and Tibshirani 1993, Huang and Chi 2012). The paired Bootstrap test based on confidence limits was used to compare the differences between treatments (Wei et al. 2020). Data of maize characteristics were checked for normality and homogeneity of variances using the Shapiro–Wilk and Levene’s test, respectively, at *P* = 0.05. The data were analyzed using one-way ANOVA, and means were separated by Tukey–Kramer (HSD) test at *P* = 0.05 using SPSS software version 16.0 (IBMCorp. 2007).

## Results

### The Biological Characteristics and Life Table Parameters of *P. interpunctella* on Different Maize Varieties

The duration of different life stages and adult longevity of *P. interpunctella* are presented in Table 1. The egg developmental duration was significantly longer on the BC678, BK, and Simon varieties than on Valbom. There were significant differences in the larval duration period among the maize varieties. The duration of larval period was significantly longer on Valbom and shortest on Simon and 703. The durations of *P. interpunctella* pupae reared on BK was significantly longer than the other tested varieties. The preadult duration was significantly longer on BK and Valbom. The oviposition period was signiﬁcantly affected by maize varieties, and the most prolonged oviposition period was obtained on the Valbom. The total preoviposition time, time from birth to ﬁrst reproduction (TPOP) in females of *P. interpunctella* reared on Valbom and BK was significantly higher than the other varieties. The most prolonged mean male longevity was observed on the males reared on 703 and Valbom. The most extended mean female longevity was obtained in the females reared on Valbom. The longest total developmental duration was related to *P. interpunctella* reared on Valbom (Table 1).

**Table 1.** Mean (±SE) development time (d) of various life stages of *Plodia interpunctella* on different maize varieties

	Variety				
**Development stage**	**Simon**	**Valbom**	**703**	**BK**	**BC678**
Egg	6.5 ± 0.1a	6.0 ± 0.1c	6.3 ± 0.1b	6.5 ± 0.1a	6.5 ± 0.1a
Larvae	21.5 ± 0.2c	24.9 ± 0.3a	20.6 ± 0.2c	22.8 ± 0.2b	22.3 ± 0.3b
Pupae	7.6 ± 0.1c	12.1 ± 0.3b	12.3 ± 0.2b	13.9 ± 0.4a	8.2 ± 0.3c
Preadult	35.5 ± 0.3d	43.1 ± 0.4a	39.2 ± 0.3b	43.4 ± 0.4a	36.8 ± 0.4c
Oviposition period	8.0 ± 0.2b	9.8 ± 0.3a	8.3 ± 0.3b	7.9 ± 0.3b	8.2 ± 0.2b
Preoviposition period (TPOP)	35.4 ± 0.4d	44.2 ± 0.5a	39.2 ± 0.4b	44.1 ± 0.5a	37.3 ± 0.6c
Adult longevity (male)	7.1 ± 0.2c	10.0 ± 0.3a	10.7 ± 0.4a	9.4 ± 0.3b	7.7 ± 0.3c
Adult longevity (female)	8.9 ± 0.3b	10.8 ± 0.3a	8.6 ± 0.4b	8.7 ± 0.3b	8.8 ± 0.2b
Total longevity	41.8 ± 0.6c	52.2 ± 0.7a	49.0 ± 0.4b	49.3 ± 0.9b	43.0 ± 0.8c

Standard errors were estimated by using the bootstrap technique with 100,000 resampling. Means followed by the same letter within a row are not significantly different using the paired bootstrap test based on the confidence interval of 100,000 differences (*P* < 0.05).

There was a significant difference in the fecundity among maize varieties, which was highest on Valbom. The maize varieties significantly affected the gross reproductive rate (GRR). The highest GRR was on BC678 and Valbom, while the lowest was on 703. The population parameter, net reproductive rate (*R*_0_) was not significantly different among maize varieties. However, a significant difference was observed in the intrinsic rate of population increase among different maize varieties. The population reared on BC678, and Simon had a higher intrinsic rate of increase (*r*), and those reared on the Valbom and BK had a lower one. The finite rate of increase (*λ*) of *P. interpunctella* was signiﬁcantly different depending on the maize varieties on which they were reared. The highest finite rate of increase was obtained when reared on BC678 and Simon, while the lowest was observed on BK and Valbom. The shortest mean generation time was found on Simon, while the longest was on Valbom (Table 2).

**Table 2.** Mean (±SE) of the fecundity and life table parameters of *Plodia interpunctella* on different maize varieties

Parameters	Variety				
	Simon	Valbom	703	BK	BC678
Fecundity (eggs/female)	110.54 ± 2.61b	132.23 ± 3.79a	115.07 ± 4.71b	115.52 ± 4.16b	118.56 ± 3.34b
GRR (offspring)	99.102 ± 17.92ab	175.645 ± 40.01a	64.761 ± 10.21b	94.999 ± 12.33ab	221.471 ± 58.69a
*R*_0_ (offspring)	40.900 ± 5.42	56.860 ± 6.76	49.480 ± 6.03	48.520 ± 5.96	54.540 ± 6.10
*r* (d^−1^)	0.094 ± 0.004a	0.083 ± 0.003b	0.091 ± 0.003ab	0.082 ± 0.003b	0.098 ± 0.003a
*λ* (d^−1^)	1.099 ± 0.004a	1.086 ± 0.003b	1.095 ± 0.004ab	1.085 ± 0.003b	1.103 ± 0.003a
*T* (d)	39.293 ± 0.38d	48.802 ± 0.53a	43.059 ± 0.59b	47.499 ± 0.58a	40.740 ± 0.38c

Standard errors were estimated by using the bootstrap technique with 100,000 resampling. Means followed by the same letter within a row are not significantly different using the paired bootstrap test based on the confidence interval of 100,000 differences (*P* < 0.05). Where no letters exist, no significant differences were noted.

**Fig. 1.**
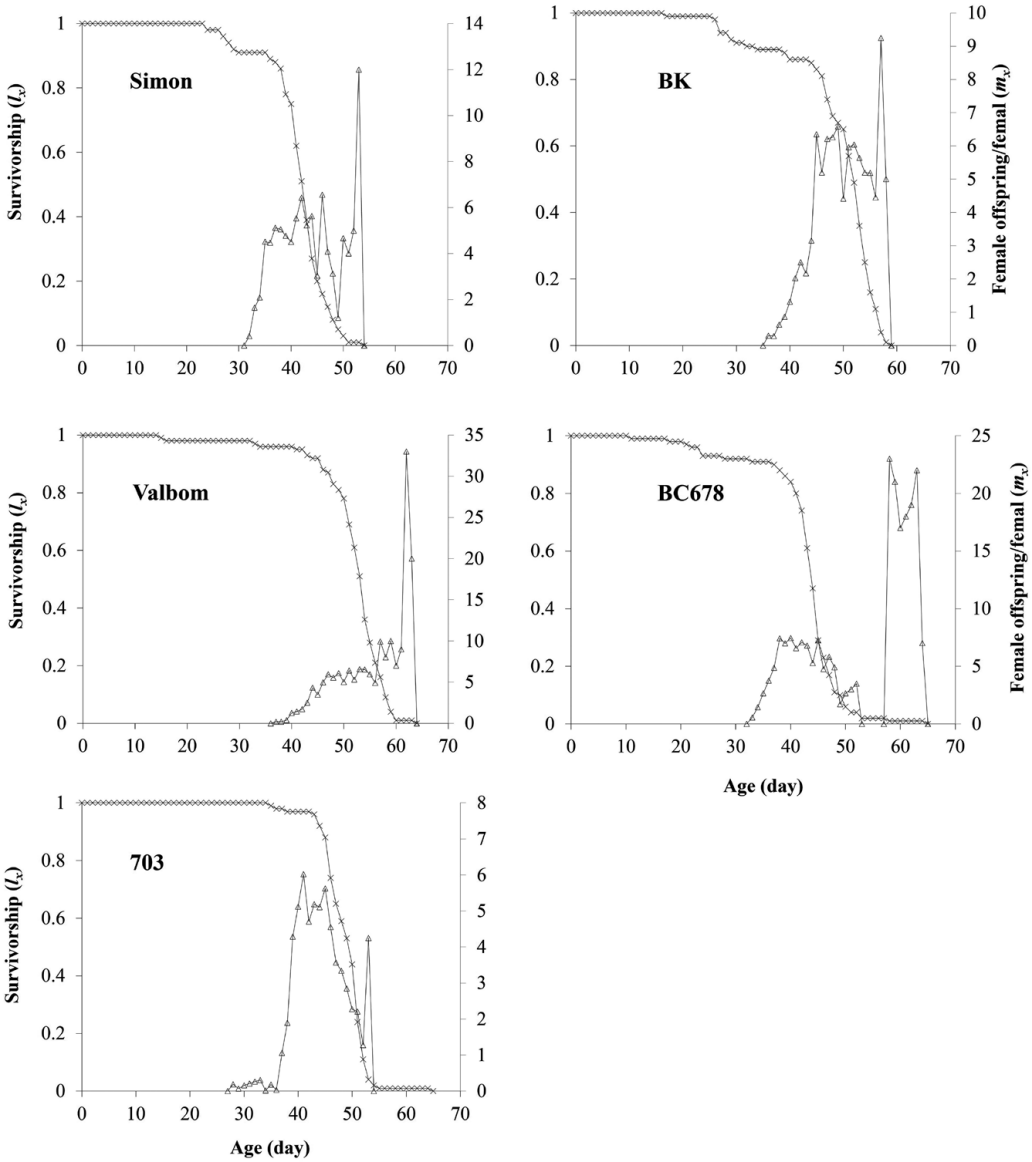
Age-specific survival rate (*l*_*x*_) and female age-specific fecundity (*m*_*x*_) of Plodia interpunctella on different maize varieties.

The age-speciﬁc survivorship curves (*l*_*x*_) of *P. interpunctella* are presented in Fig. 1. The highest and lowest survival rates of preadult stages that survived to the adult stage were found when reared on 703 and BC678, respectively. The death of the last female varied from 65 d when reared on 703 to 54 d on Simon. The age-specific fecundity (*m*_*x*_) of *P. interpunctella* is shown in Fig. 1. The number of laid eggs at the peak of oviposition of females were 12.0, 33.0, 6.0, 9.3, and 7.5 eggs per female per day on Simon, Valbom, 703, BK and BC678, respectively (Fig. 1).

**Fig. 2.**
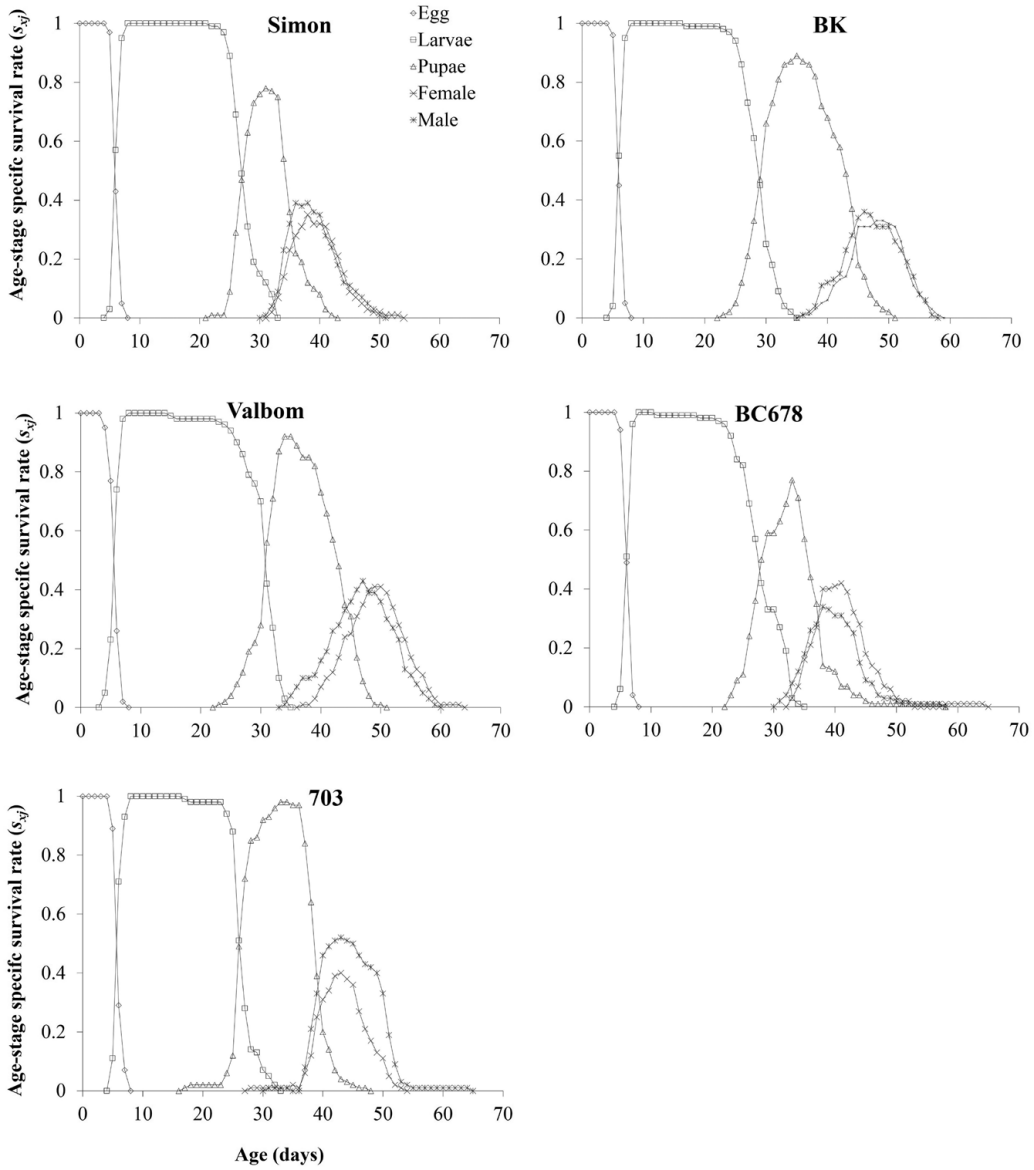
Age–stage specific survival rate (*s*_*xj*_) of *Plodia interpunctella* on different maize varieties.

Age–stage specific survival rates (*s*_*xj*_) of *P. interpunctella* are shown in Fig. 2, indicating the probabilities that a newborn larva will survive to age *x* and stage *j* on different maize varieties. The *s*_*xj*_ curve for *P. interpunctella* females was higher on Valbom, 703, and BC678, reflecting the higher preadult survival rates on these hosts. The *s*_*xj*_ for males was higher in the populations reared on 703 (Fig. 2).

**Fig. 3.**
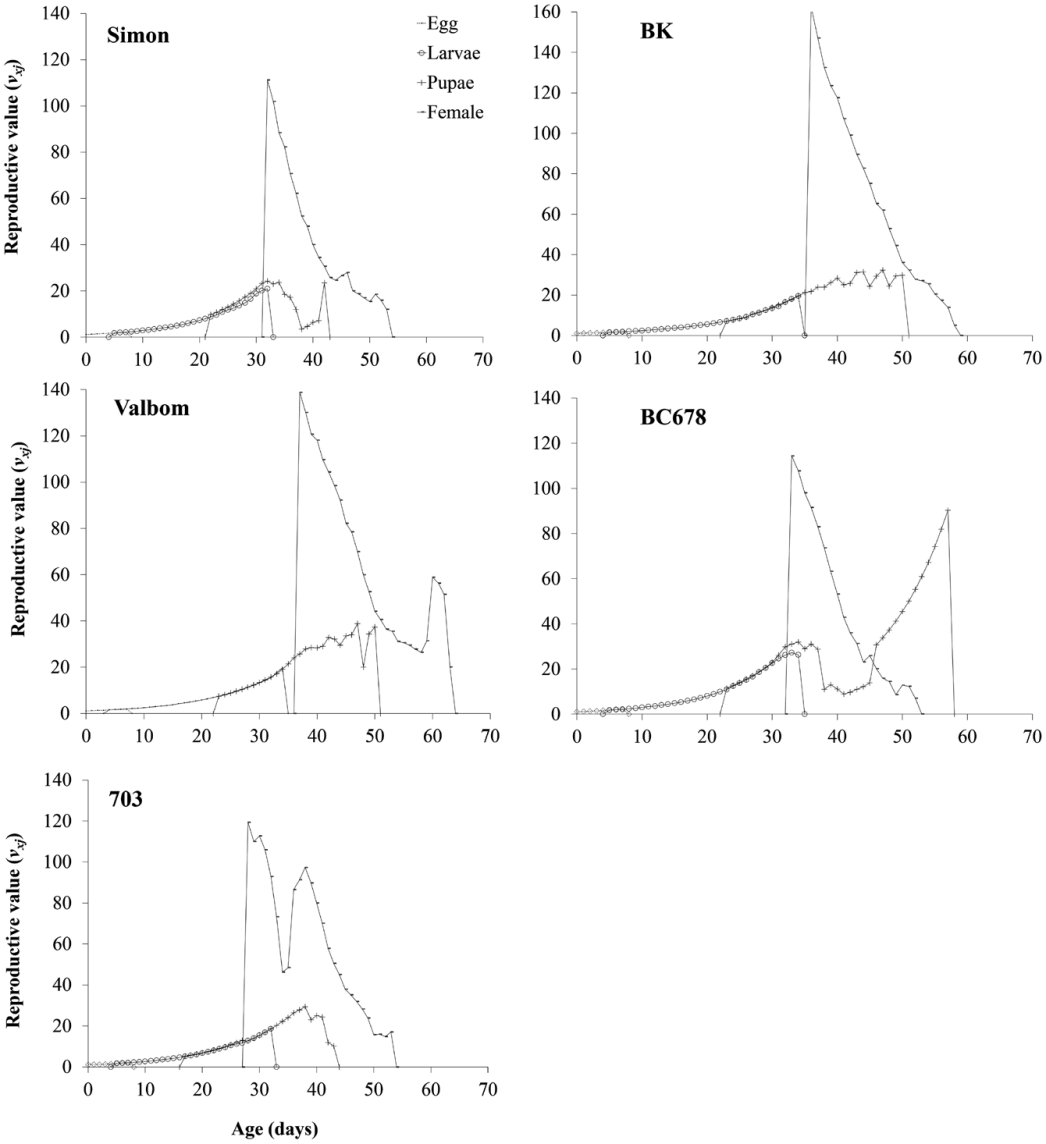
Age–stage specific reproductive value (*v*_*xj*_) of *Plodia interpunctella* on different maize varieties.

The reproductive value (*v*_*xj*_) for each age–stage group is presented in Fig. 3, indicating the expected contribution of an individual of age *x* and stage *j* to the future population. The major reproductive curve peaks for individuals reared on Simon, Valbom, 703, BK, and BC678 occurred at 32 (111.2 d^−1^), 37 (138.8 d^−1^), 28 (119.4 d^−1^), 37 (147.1 d^−1^), and 34 d (114.3 d^−1^), respectively. The peak reproductive values on 703 and Simon occurred earlier than the other varieties (Fig. 3).

**Fig. 4.**
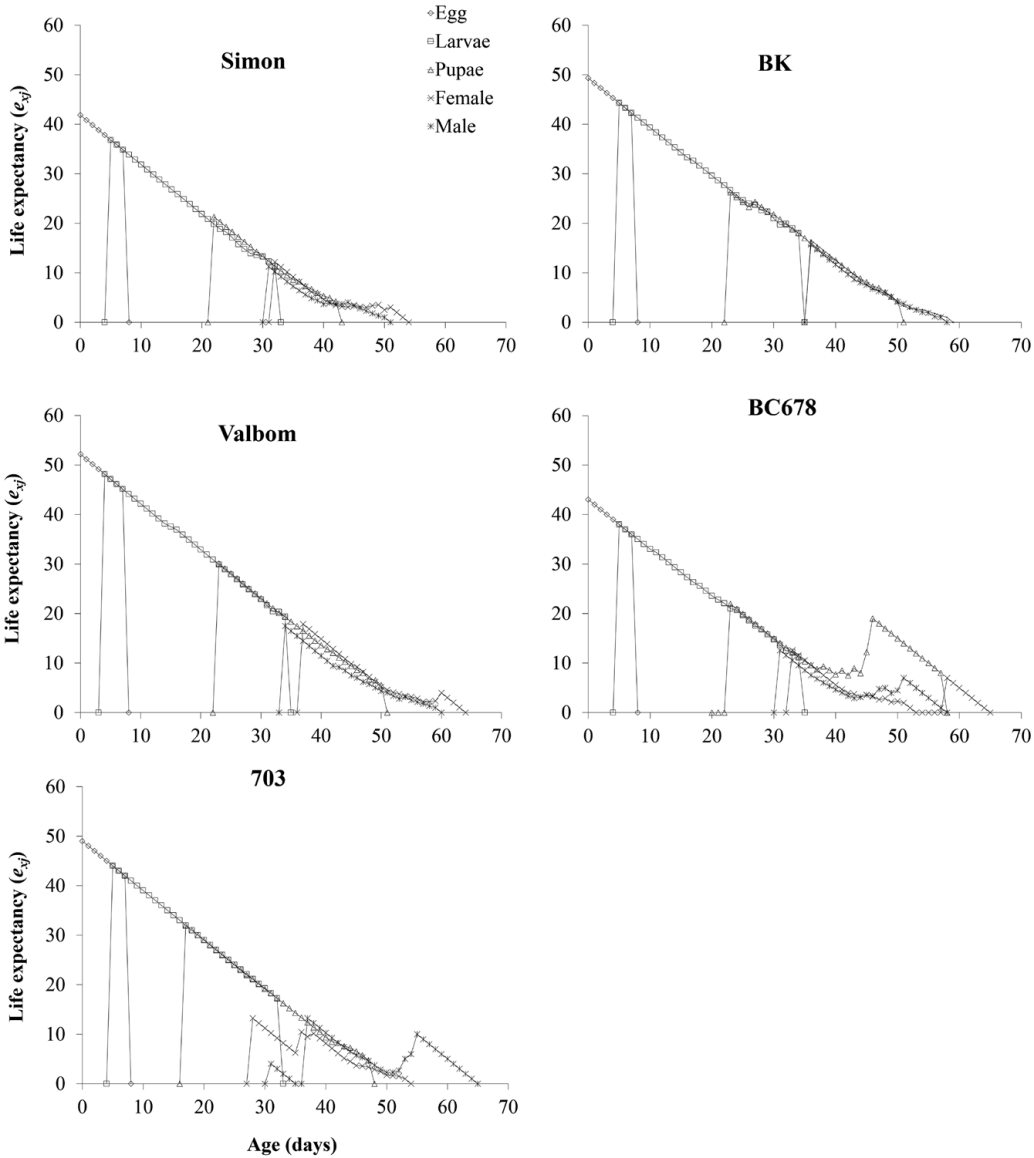
Age–stage specific life expectancy (*e*_*xj*_) of *Plodia interpunctella* on different maize varieties.

The life expectancy (*e*_*xj*_) for each age–stage group reared on different maize varieties is presented in Fig. 4, indicating the expected length of time that an individual of age *x* and stage *j* will survive. The life expectancy of an egg was 34.8, 45.2, 42.0, 42.3 and 36.0 d on Simon, Valbom, 703, BK and BC678, respectively (Fig. 4).

### Characteristics of Maize Kernels

The moisture content of maize varieties is presented in Table 3. The moisture content of maize kernels ranged from 7.8% to 9.0%. The highest moisture content was observed in Simon, followed by BC678, while the lowest was in Valbom (*F*_4,10_ = 63.77; *P* < 0.001). The protein content significantly differed among tested maize kernels (*F*_4,10_ = 5.57; *P* = 0.013). The highest protein content was recorded in the BC678 and Simon, while the lowest in BK. The starch content of maize kernels significantly differed among varieties. The starch content was significantly higher in the BC678 compared to the other tested varieties (*F*_4,10_ = 5.57; *P* = 0.013) (Table 3).

**Table 3.** Mean (±SE) of moisture content percentage, protein and starch content in different maize varieties

Characteristics	Variety				
**Simon**	**Valbom**	**703**	**BK**	**BC678**
Moisture content (%)	9.0 ± 0.1a	7.8 ± 0.0c	8.3 ± 0.1b	8.2 ± 0.1b	8.8 ± 0.1a
Protein (mg/g)	96.7 ± 1.3a	89.9 ± 1.3ab	94.3 ± 0.1ab	83.6 ± 0.4b	98.8 ± 5.4a
Starch (mg/g)	481.6 ± 1.3c	652.1 ± 1.6b	382.4 ± 3.8d	348.3 ± 3.5e	677.0 ± 2.1a

Within each row, means followed by the same letter are not significantly different by Tukey–Kramer (HSD) test at *P *< 0.05.

## Discussion

The results on the influence of maize varieties on the biological traits and life history parameters of *P. interpunctella*, provides valuable insights into pest management strategies in agricultural settings. The research demonstrated significant variations in the developmental duration of *P. interpunctella*, which ranged from 31 to 35 d, depending on the moisture content of the 5 tested maize varieties. This finding underscores the importance of considering both genetic and environmental factors when assessing pest biology and development. One of the key findings was that the longevity of *P. interpunctella* was notably shorter when reared on maize varieties such as Simon (41.8 d) and BC678 (43.0 d), which exhibited higher moisture content. This suggests that high moisture levels in certain maize varieties can accelerate the consumption and growth of *P. interpunctella*, making these kernels more susceptible to infestation. Conversely, lower moisture content in the Valbom variety resulted in a prolonged developmental duration and increased longevity for the moths, indicating that drier kernels may be less conducive to pest proliferation. Our study aligns with previous research indicating that high moisture content in grains is advantageous for *P. interpunctella* larvae, emphasizing the need for moisture management in grain storage and handling. Johnson et al. (1992) noted that varieties with high moisture content were more suitable for *P. interpunctella* larval development. The larval growth rates in wheat germs increased with an increase in the water content of the cereal diets (Silhacek and Murphy 2008). These results can inform agricultural practices by guiding farmers in selecting maize varieties that are less favorable for *P. interpunctella* development. Cleaning and drying grains prior to storage, farmers can partially prevent larvae from entering the grain, reduce infection rates, and minimize losses (Makinya et al. 2021). Understanding the developmental preferences of *P. interpunctella* can help determine pest management strategies in different stored products For instance, commodities like wheat flour, sorghum, and barley were found to be less suitable for *P. interpunctella* development due to their nutritional composition and moisture levels (Bouayad et al. 2008).

In addition, the development and fecundity of *P. interpunctella* is influenced by the maize kernels mechanical state and type (Predojević et al. 2017). Also, smaller-sized kernels are least favorable due to the inability of newly hatched larvae to enter undamaged kernels (Abdel-Rahman et al. 1968). Other factors such as environmental conditions (including temperature and RH), oviposition opportunities, water presence and food for consumption could also influence longevity of *P. interpunctella* (Gvozdenac et al. 2018). In this study, the highest number of eggs were laid on Valbom (132.2 eggs per female), while there were no significant differences among other maize varieties. Fecundity of *P. interpunctella* could also depend on the mechanical characteristics of kernels. Broken and dented maize kernels were more appropriate for *P. interpunctella* egg-laying than the ground ones (Predojević et al. 2017). On the other hand, soft kernels with floury endosperm were more suitable for *P. interpunctella* larval consumption, require less energy for breaking the kernel pericarp, and lead to more oviposition (Gvozdenac et al. 2018). Our results indicate that BK was less suitable host plants to *P. interpunctella*.

In this research, the highest GRR (eggs/individual) on BC678 and Valbom could be attributed to the high starch content in these varieties. Predojević et al. (2017) noted that softest kernel types with higher percentage of ﬂoury endosperm are more prone to consume and egg-laying. In contrast, hard outer layer with a low starch content in Popcorn makes them unfavorable for larval development (Gvozdenac et al. 2018). The intrinsic rates of increase were significantly different concerning the maize varieties. The highest protein content in the BC678 and Simon led to an increase in the number of offspring/day (intrinsic rate of increase). However, the lowest intrinsic rate of increase on Valbom and BK indicating unsuitability of these varieties to *P. interpunctella,* as time approaches infinity and the population settles down, and this is in stable age–stage distribution. Mutant kernels without embryos were less damaged and showed the lowest mean number of emerged *P. interpunctella* adults (Limonta et al. 2013). Differences among nutritional content of the food commodity could affect the biological performance of an insect species. The high *rm* value on Akbari cultivar among the 4 tested pistachio varieties can be attributed to that this host is more suitable for the development and reproduction of *P. interpunctella* (Razazzian et al. 2015). Therefore, biochemical compounds in food commodities have an outstanding effect on insect growth and survival. Walnuts contain large amounts of polyunsarurated fatty acids, and oxidative rancidity can make them unsuitable to *P. interpunctella* (Johnson et al. 1992).

Our results showed that the biological traits of *P. interpunctella* can be influenced by biochemical characteristics and type of maize kernels. The presence of specific biochemical compounds in different maize varieties plays a crucial role in the growth and survival of *P. interpunctella*, leading to notable variations in its performance. Moreover, the research suggests that certain maize varieties exhibit less suitable for *P. interpunctella*, particularly those with lower intrinsic rates of increase. Therefore, understanding the biological interactions between *P. interpunctella* and various maize varieties can help in setting a well-documented program for its effective control. Future research should continue to explore these relationships to enhance strategies to control *P. interpunctella* populations in agricultural settings.
